# Till Death Do Us Part—The Multifaceted Role of Platelets in Liver Diseases

**DOI:** 10.3390/ijms22063113

**Published:** 2021-03-18

**Authors:** Marion Mussbacher, Laura Brunnthaler, Anja Panhuber, Patrick Starlinger, Alice Assinger

**Affiliations:** 1 Department of Pharmacology and Toxicology, University of Graz, 8010 Graz, Austria; marion.mussbacher@uni-graz.at; 2 Center of Physiology and Pharmacology, Medical University of Vienna, 1090 Vienna, Austria; laura.brunnthaler@meduniwien.ac.at (L.B.); anja.panhuber@meduniwien.ac.at (A.P.); 3 Department of Surgery, Medical University of Vienna, General Hospital, 1090 Vienna, Austria; patrick.starlinger@meduniwien.ac.at; 4 Department of Surgery, Division of Hepatobiliary and Pancreas Surgery, Mayo Clinic, Rochester, MN 55905, USA

**Keywords:** platelets, liver, non-alcoholic fatty liver disease, NASH, HCC, liver regeneration

## Abstract

Platelets are tightly connected with the liver, as both their production and their clearance are mediated by the liver. Platelets, in return, participate in a variety of liver diseases, ranging from non-alcoholic fatty liver diseases, (viral) hepatitis, liver fibrosis and hepatocellular carcinoma to liver regeneration. Due to their versatile functions, which include (1) regulation of hemostasis, (2) fine-tuning of immune responses and (3) release of growth factors and cellular mediators, platelets quickly adapt to environmental changes and modulate disease development, leading to different layers of complexity. Depending on the (patho)physiological context, platelets exert both beneficial and detrimental functions. Understanding the precise mechanisms through which platelet function is regulated at different stages of liver diseases and how platelets interact with various resident and non-resident liver cells helps to draw a clear picture of platelet-related therapeutic interventions. Therefore, this review summarizes the current knowledge on platelets in acute and chronic liver diseases and aims to shed light on how the smallest cells in the circulatory system account for changes in the (patho)physiology of the second largest organ in the human body.

## 1. Introduction

**Figure 1:**
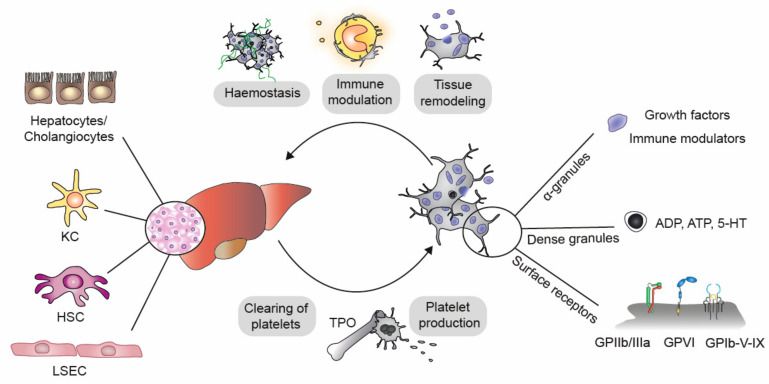
Interactions between liver and platelets. The liver consists of parenchymal (90% hepatocytes) and non-parenchymal cells (2–3% cholangiocytes, 2.5% liver sinusoidal endothelial cells [LSEC], 2% Kupffer cells [KC] and 1.4% hepatic stellate cells [HSC]) [7]. Parenchymal cells secrete thrombopoietin (TPO), which stimulates platelet production and maturation. The liver is further involved in platelet clearance. Platelets modulate liver functions by the release of α- and dense granules, which contain growth factors and immune modulators as well adenosine diphosphate (ADP), adenosine triphosphate (ATP) and serotonin (5-HT), respectively. Platelet surface receptors (GPIIb/IIIa, GPVI, GPIb-V-IV) allow interactions with hepatic cells and extracellular matrix components, which initiates tissue remodeling and hemostasis and promotes immune modulation.

Platelets have a close and lifelong relationship with the liver, the largest gland and the second largest organ in the human body (Figure 1). Liver parenchymal cells and sinusoidal endothelial cells constantly synthetize the glycoprotein hormone thrombopoietin (TPO), the key regulator of megakaryopoiesis and thrombopoiesis, which promotes the formation of long, cytoplasmic megakaryocyte expansions and the subsequent fission of platelets into the blood stream [1]. The liver thereby fosters the generation of 100 billions of anucleated, cytoplasmic cell fragments from megakaryocytes in a tightly regulated process. The importance of liver-derived TPO is evident from the strong association of severe liver diseases with thrombocytopenia, which is defined by a platelet count below 150 × 10^9^/L [2]. Moreover, the prevalence and severity of thrombocytopenia correlates with the stage of liver disease [3], and longitudinal changes in platelet counts are used as surrogate markers for certain liver diseases [4]. As platelets are essential for blood loss prevention by patrolling and sealing the vasculature, patients with liver pathologies often suffer from an increased bleeding risk and often require platelet transfusions. Due to the limited life span of platelets, which ranges from 7 to 10 days in humans, repeated platelet transfusions are often necessary. However, this bears the risks of associated alloimmunization and other transfusion-associated complications, which can be overcome by TPO receptor agonists, like elthrombopaq, which stimulate platelet production.

Liver transplantation results in thrombocytopenia, which stabilizes approximately after 14 days in most patients, and immediate low postoperative platelet counts represent an independent predictor of delayed liver function recovery and increased post-operative mortality, while platelet transfusions improve the outcome [5,6]. This indicates not only that the liver guards the fate of platelets but also that platelets are crucial for liver homeostasis.

## 2. Mechanisms of Interaction between Platelets and Liver

While the liver secures platelet production, platelets maintain liver homeostasis via three major strategies: First, platelets promote hemostasis and vascular integrity—a prerequisite for sufficient blood flow through the complex network of the liver vasculature, which requires high oxygen support for amino acid and lipids metabolism. Second, platelets fine tune the immune system by direct and indirect interactions with both innate and adaptive immune cells. This is essential to protect the liver from pathogens, on the one hand, but also propagates harmful inflammatory responses, on the other hand. Last, platelets carry a large reservoir of biologically active substances like growth factors, including the vascular endothelial growth factor (VEGF), hepatocyte growth factor (HGF) and platelet-derived growth factor (PDGF), and inflammatory molecules like chemokine (C-X-C motif) ligand 1 (CXCL) 1, CXCL4, CXCL5 and CXCL7. These molecules are site-specifically released to support liver parenchymal and non-parenchymal cell functions (Figure 1).

Upon injury, platelets adhere via the binding of their glycoprotein (GP) receptors GPIb-V-IX and GPVI to the immobilized von Willebrand factor (vWF) and subendothelial collagen. Subsequent platelet activation leads to a conformational change of GPIIb/IIIa, allowing platelets to bind to fibrinogen, which bridges platelet-platelet interactions and thrombus formation. Upon adhesion, platelets release their granule content, which contains a plethora of downstream effector molecules that guide hemostatic, immunological and tissue remodeling processes. In the liver little is known on the precise interactions that trigger platelet adhesion processes during liver diseases.

The activation of platelets is observed in a broad range of liver diseases, indicating that platelets act as fast-responding sentinels scanning the liver microenvironment and responding to vascular injury [8,9]. Moreover, the liver synthesizes acute-phase proteins, coagulation factors and lipoproteins, which activate platelets under specific conditions, thereby adding additional layers of complexity via feedback mechanisms.

Within the liver, platelets interact with the vWF expressed on Kupffer cells via GPIb, which is important for the so-called “touch-and-go” maneuvers that mediate hepatic immunosurveillance [6]. Platelets migrate to sites of inflamed liver sinusoids to scavenge fibrin-bound bacteria and to modulate immune responses via direct and indirect interactions.

Upon activation, platelets interact with leukocytes—preferentially neutrophils and monocytes—thereby assisting in their activation, recruitment and a variety of effector functions. Platelet-leukocyte interaction is mediated by direct cell-to-cell contact via P-selectin binding to P-selectin binding glycoprotein 1 (PSGL-1) and/or indirectly via the release of platelet granule content, which contains chemokines and cytokines. In several liver diseases platelet-leukocyte aggregates are increased [10], which is either beneficial or detrimental depending on the type and stage of liver diseases.

If not trapped within a platelet plug, the life of a platelet ends in the reticuloendothelial system of the liver or spleen and is precisely controlled by an intrinsic apoptotic balance, which includes pro-survival Bcl-xL and pro-apoptotic Bak signals [11]. This process is facilitated by platelet surface changes, mainly via increased phosphatidyl serine (PS) and loss of sialic acid residues [12], which allows binding to the lectin asialoglycoprotein receptor (ASGPR) on hepatocytes and Kupffer cells and serves as an ingestion signal for platelets [13].

## 3. Liver Diseases and Their Impact on Platelet Count

Non-alcoholic fatty liver disease (NAFLD) is an umbrella term for a variety of gradually developing fatty liver diseases with a worldwide prevalence of 25%, that reaches up to 75% in the obese population [14,15]. Due to its strong association with obesity and diabetes, NAFLD is often considered as the hepatic manifestation of the metabolic syndrome, and treatment options are mainly limited to lifestyle changes.

**Figure 2:**
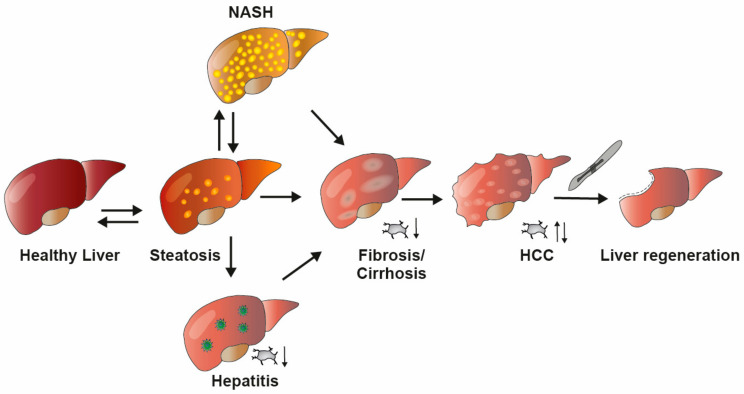
Overview of liver diseases and their impact on platelet count. Metabolic disturbances promote the development of non-alcoholic fatty liver (NAFL) disease, which is characterized by the intrahepatic accumulation of (toxic) lipids species. In general, NAFLD is reversible; however, it eventually progresses into non-reversible non-alcoholic steatohepatitis (NASH). In viral hepatitis, hepatic cells, immune cells and platelets work in concert to fight viral infections which also foster tissue damage. Hepatitis is associated with decreased platelet counts and can, like NASH, progress to hepatic fibrosis, which is hallmarked by an excessive production and deposition of extracellular matrix proteins and associated with a decrease in platelet count and leads to liver cirrhosis. This pro-carcinogen environment further promotes the development of hepatocellular carcinoma (HCC), which is characterized by both elevated and decreased levels of platelets. Hepatectomy represents a curative treatment of HCC, which relies on liver regeneration.

From a pathophysiological point of view, NAFLDs encompass a broad spectrum of gradually developing liver diseases fluently transitioning into each other (Figure 2) [16]: Initial events such as metabolic dysbalances including obesity and type two diabetes (T2D) lead to the accumulation of (toxic) lipid species, gradually developing from non-inflammatory steatosis to hepatitis (non-alcoholic steatohepatitis; NASH), which represents a unique pro-inflammatory microenvironment due to the activation of both infiltrating and resident immune cells [17]. This chronic inflammatory state eventually leads to the destruction of the liver parenchyma (hepatocyte damage) and the subsequent transformation into fibrous tissue (liver fibrosis and liver cirrhosis) [18]. Limited TPO production due to decreased liver function in fibrosis and (viral) hepatitis leads to reduced platelet production in the bone marrow and subsequently decreases the platelet count. Hepatocellular stress and apoptotic cell death further contribute to genomic instability, that predisposes patients to develop liver cirrhosis and hepatocellular carcinoma (HCC). In HCC, platelet production is either decreased due to liver dysfunction or—in some rare cases—enhanced via TPO-producing cancer cells, and platelet counts often correlate with HCC progression. While a beneficial effect of antiplatelet therapy in HCC remains to be elucidated, current curative treatments for HCC involve hepatectomy, which is followed by liver regeneration or liver transplantation.

## 4. Hepatic Steatosis (NAFLD)

### 4.1. Procoagulatory and Prothrombotic Events at the Onset of Fatty Liver Diseases

Hepatic steatosis is caused by metabolic imbalances that lead to the accumulation of (toxic) lipid species in the liver, and insulin resistance and obesity are pivotal in the pathogenesis of NAFLD. Accordingly, more than 80% of NAFLD patients in Europe and in Northern America are obese [14]. Moreover, although 5–8% are lean and are often referred to as “lean NAFDL”, these patients still have abnormal glucose tolerance and excessive abdominal adipose tissue [19].

**Figure 3:**
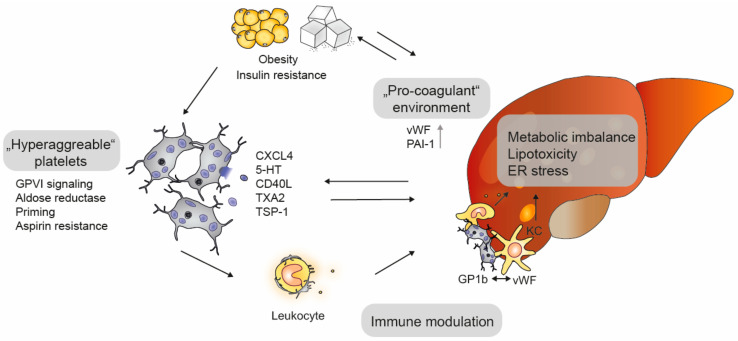
Platelets in non-alcoholic fatty liver disease (NAFLD). Obesity and insulin resistance promote the development of NAFL, which leads to a pro-coagulant state (e.g., increased von Willebrand factor [vWF] and plasminogen activator inhibitor [PAI-1]) and hyperaggregable platelets (e.g., elevated GPVI signaling, aldolase activity, platelet priming and aspirin resistance). The interaction with leukocytes and the release of granule content by activated platelets either promote (CD40 ligand [CD40L], chemokine (C-X-C motif) ligand [CXCL4] and serotonin [5-HT], thromboxane A2 [TxA2]) or prevent (thrombospondin-1 [TSP-1]) NAFLD progression.

Platelets are both victims and culprits in the development of hepatic steatosis and are linked to both insulin resistance and obesity (Figure 3) [20]. Several studies suggest that patients that are prone to develop NAFLD have hyper-reactive platelets, as platelet aggregation in response to various agonists, including collagen, adenosine diphosphate (ADP), arachidonic acid (AA) and thromboxane A2 (TxA2), is mildly increased in obese patients [21,22]. Interestingly, even after treatment with low-dose acetylsalicylic acid (aspirin, ASA), this hyper-reactive state persists, resulting in residual platelet activity [21,23]. The adipokine leptin provides a potential link between platelets, obesity and NAFLD. Leptin levels correlate with NAFLD or NASH severity and promote arterial thrombosis in a platelet leptin receptor-dependent manner [24,25]. While leptin has no direct effect on platelet function, it enhances ADP-induced aggregation at clinically relevant concentrations [26,27]. In patients with T2D the enzyme aldose reductase potentially triggers platelet hyperreactivity. Aldose reductase becomes activated in platelets under hyperglycemia and stimulates TxA2 synthesis and release, thereby increasing platelet activation [28]. Circulating TxA2 levels as well as hepatic TxA2 receptor (TxA2R) expression are upregulated in NAFLD [29], and the blockage of TxA2 signaling with genistein effectively attenuates high fat diet (HFD)-induced steatosis and insulin resistance in experimental models. However, to date, clinical evidence is lacking, and the specificity of genistein is limited. Therefore, further studies on knock-out animals are warranted to decipher the precise role of aldose reductase in NAFLD [29].

NAFLD further results in a hypercoagulatory state with an increased thrombotic risk due to elevated levels of vWF and plasminogen activator inhibitor type I (PAI-1). However, recent studies suggest a potential “re-calibration” of the hemostatic status in NAFLD patients [30] and point to additional mechanisms that account for their increased risk of venous thromboembolism [31] and portal vein thrombosis [32]. Nonetheless, hyperfibrinolysis and changes in fibrin clot structures occur in NAFLD and correlate with the increased glycation and oxidation of fibrinogen [33,34]. Fibrin may promote NAFLD development by mechanisms beyond hemostasis involving intravascular inflammation and the formation of hepatic microthrombi [35].

The activated coagulation cascade in NAFLD leads to thrombin generation, which not only cleaves fibrinogen into fibrin, but also represents the strongest platelet activator via proteinase activated receptor 1–4 (PAR1–4) signaling. This further fosters platelet hyper-reactivity in NAFLD. While experimental models confirm thrombin generation in NAFLD [36], clinical evidence is lacking. Thrombin inhibition with dabigatran and mouse models with reduced tissue factor (TF) or PAR1 knockout ameliorates HFD-induced obesity and NAFLD [36,37]. However, further studies are warranted to pinpoint the precise involvement of primary and secondary hemostasis in NAFLD and to translate the results into clinical settings.

### 4.2. The Role of Platelet-Derived Molecules in Fatty Liver Diseases

Upon activation, platelets release their granule content containing growth factors, inflammatory mediators and coagulation factors. Platelet granule-derived molecules are increased in both human and mouse NAFLD. One important protective platelet-derived mediator in NAFLD is thrombospondin (TSP-1). Exogenous TSP-1 strongly ameliorated hepatic steatosis due to the CD36-dependent inhibition of lipogenic gene expression in a mouse model of diet-induced obesity [38]. However, as TSP-1 can also be produced by Kupffer cells, adipocytes, endothelial cells and hepatic stellate cells [38], the contribution of platelets in this process remains to be elucidated.

Upon activation, CD40L, a member of the tumor necrosis factor (TNF) superfamily, which is almost selectively released from platelets, is exposed and shed from the platelet surface, allowing for interaction with CD40-expressing leukocytes. Soluble CD40L levels are increased in NAFLD patients [39], and peripheral blood gene sequencing showed that CD40/CD40L signaling was one of the four major pathways upregulated in individuals with fatty liver [40]. In line with these findings, mouse studies using either CD40L knockout mice or αCD40L blocking antibodies showed attenuated development of diet-induced obesity, hepatic steatosis and increased insulin sensitivity [41]. Interestingly, CD40L plays a protective role in regulating lipid processing in the liver. CD40L knockout mice exert impaired very low density lipoprotein (VLDL) secretion and an increased hepatic expression of lipogenic genes upon feeding with an olive oil-rich diet in the absence of inflammation [42]. This makes CD40L a double-edged sword in NAFLD, which, depending on the underlying inflammatory state, exerts beneficial or detrimental effects.

The most abundant platelet-released protein is CXCL4, which binds glycosaminoglycans and the chemokine receptor CXCR3 [43]. Although no studies on CXCL4 in the development of NAFLD are available, data on CXCR3 indicate an involvement in lipid accumulation and endoplasmic reticulum (ER) stress in mouse models of steatohepatitis [44].

Another important platelet-derived mediator is 5-HT [45]. The genetic inhibition of gut-derived 5-HT synthesis renders mice resistant to HFD-induced hepatic steatosis without affecting their systemic energy balance [46]. However, further work is necessary to pinpoint the precise role of platelets in delivering 5-HT.

### 4.3. Other Factors Associated with Fatty Liver Disease

Compelling evidence from mouse studies demonstrates an important role of the microbiome in the transition from NAFLD to its severe form, NASH [47]. As gut bacteria either directly or indirectly provide important signaling mediators that regulate bile acid synthesis, lipid and glucose metabolism, inflammatory processes as well as intestinal permeability [16], it is plausible that platelets get activated under these conditions and exert disease-promoting functions. Recently, a new gut microbial metabolite, phenylacetylglutamine (PAPln), has been identified in an untargeted metabolomic approach. PAPln promotes platelet hyperreactivity via binding to adrenergic receptors [48], and the gut microbe metabolite trimethylamine N-oxide (MAO) causes platelet hyperreactivity [49]. Moreover, cirrhotic patients show increased levels of lipopolysaccharide (LPS) and the gut permeability marker zonulin, which correlate with increased platelet reactivity. Low-dose endotoxemia might explain the pro-thrombotic state of cirrhosis patients, which is associated with an increased risk of bleeding due to hyper-fibrinolysis and thrombocytopenia. However, low-dose LPS alone could not recapitulate the platelet responses observed during endotoximia [50], indicating a more complex interplay of platelets and hepatic cells. As platelets express Toll-like receptors (TLRs), allowing for pathogen interaction [51], and regulate gut permeability by 5-HT release, they likely play an important “bridging” function between the gut and the liver.

## 5. Hepatitis: Steatohepatitis and Viral Hepatitis

**Figure 4:**
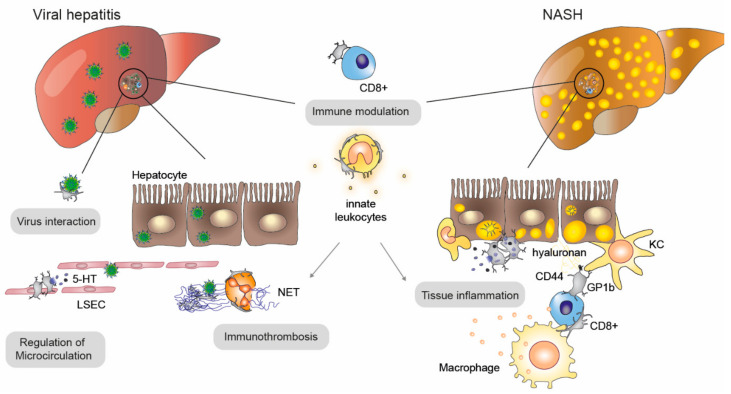
The role of platelets in viral hepatitis and NASH. Platelets become activated in non-alcoholic steatohepatitis (NASH) and viral hepatitis and contribute to disease progression by several mechanisms: recruitment of leukocytes (e.g., CD8+ T cells) via direct and indirect interactions, which fosters immune modulation but also immunothrombosis (e.g., by the promotion of neutrophil extracellular trap (NET) formation by neutrophiles) and tissue inflammation. The interaction of platelets with liver-resident Kupffer cells (KC) is mediated by CD44-hyaluronan as well as von Willebrand Factor (vWF)-GP1b, which is important for immunosurveillance. Platelets directly interact with viruses to either shelter or present them to immune cells. Another important platelet-mediated mechanism is the regulation of local blood flow in liver sinusoids by the release of serotonin (5-HT), which acts on liver sinusoidal endothelial cells (LSECS).

Platelets are detected in the steatotic liver prior to the infiltration of immune cells and subsequent progression of NAFLD into its severe form, NASH, indicating a contribution of platelets to leukocyte recruitment (Figure 4). In line with these findings, platelet depletion and antiplatelet therapy reduce hepatic inflammatory infiltrates in a diet-induced mouse model of NASH [9]. The interaction of platelets with Kupffer cells is mediated by platelet CD44’s binding to hyaluronan of the extracellular matrix [9], and effector CD8 cells interact via platelet CD44-hyaluronan to fulfill the immunosurveillance within liver sinusoids during a hepatitis virus infection [52]. Platelets accelerate inflammation mainly via α‑granule release and interaction with immune cells via GP1b. A recent prospective study of NAFLD patients showed that the daily use of aspirin was associated with lower incidences of NASH and fibrosis [53]. Interestingly, hyper-reactive platelets with impaired granule secretion were found in patients with severe alcoholic hepatitis due to increased levels of oxidized plasma albumin [54], which induces platelet activation in a CD36-dependent way [54], promoting inflammation and apoptosis in immune-mediated hepatitis [55].

Viral hepatitis is frequently associated with reduced platelet counts caused by the destruction of liver parenchymal cells as well as decreased TPO production. Platelets are often activated in viral hepatitis and are in close contact with periportal infiltrates of CD8+ cells, indicating an active role of platelets in the pathophysiology of hepatitis [56]. Platelets can modulate (viral) hepatitis at different levels: (i) platelets promote the recruitment of virus-specific CD8+ cells and secondary innate immune cells, (ii) platelets locally restrict viral spreading by micro/immunothrombosis, (iii) platelets modulate sinusoidal microcirculation. Depending on the disease stage, e.g., initial infection vs. long-term tissue damage, all these functions can play either a beneficial (e.g., by preventing the spreading of the virus) or a detrimental role (e.g., by exacerbating CD8+ T cell-mediated tissue damage at later stages of the disease). In general, platelet-derived 5-HT reduces sinusoid microcirculation and increases liver damage by delaying the infiltration of virus-specific CD8+ cells [56]. Moreover, platelet depletion reduces the recruitment of CD8+ T cells and thereby ameliorates hepatocyte damage in a coagulation-independent manner [57]. Consequently, a low-dose treatment of aspirin and clopidogrel reduces the homing of cytotoxic T cells and associated liver disease severity [58]. Further, the risk for chronic hepatitis B and hepatitis C patients to suffer from liver-related mortality is significantly reduced upon long-term aspirin intake [59]. Platelets interact with neutrophils, which initiates the formation of neutrophil extracellular traps (NETs), thereby supporting viral clearance in the liver vasculature [60]. Interestingly, platelets also act as a biological reservoir of hepatitis C virus and preserve the virus from degradation [61], further pointing to the multi-facetted role of platelet-virus interactions [62].

## 6. Liver Fibrosis

**Figure 5:**
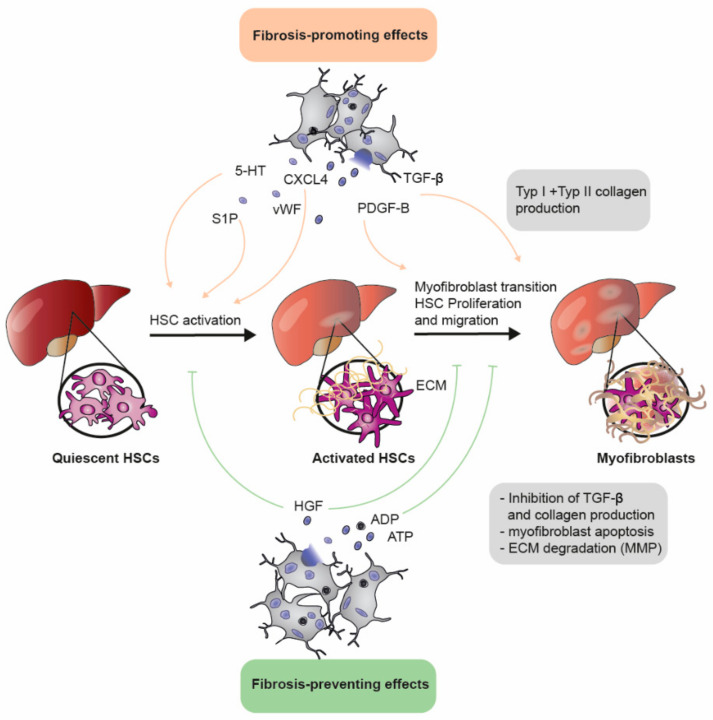
Dual role of platelets in liver fibrosis. The activation of hepatic stellate cells (HSC) leads to the excessive production of extracellular matrix (ECM) proteins, especially type I and type II collagens. This process is promoted by the release of serotonin (5-HT), sphingosine-1-phosphate (S1P) and platelet factor 4 (CXCL4) from platelet granules. Subsequently, platelet-derived growth factor B (PDGF-B) and transforming growth factor-*β* (TGF-*β*) promote the proliferation and differentiation of activated HSC into myofibroblasts. In contrast, hepatic growth factor (HGF) as well as adenosine-diphosphate (ADP) and adenosine-triphosphate (ATP) released from platelets counteract both processes and therefore play a beneficial role in liver fibrosis.

Liver fibrosis is defined by excessive hepatic production and the deposition of extracellular matrix (ECM) proteins, leading to fibrotic scars, distorted liver structure and portal hypertension caused by the subsequent increase in intrahepatic blood flow resistance. Activated hepatic stellate cells (HSCs) are the major source of ECM proteins, especially of type I and type II collagen [63], and platelets are able to regulate HSCs activation by releasing both anti- and pro-fibrotic molecules [64] (Figure 5).

Clinical studies indicate that higher platelet levels are generally associated with less liver fibrosis and that platelet transfusions improve liver functions and reduce hepatic fibrosis, especially in chronic liver disease (CLD), cirrhosis and chronic hepatitis B [65,66]. These beneficial effects are attributed to the release of anti-fibrotic molecules such as adenine nucleotides and HGF from platelet granules [67,68,69]. Mechanistically, adenine nucleotides are degraded by HSCs to adenosine, which subsequently activates adenylyl cyclase (AC) and cAMP-dependent pathways that inhibit HSC proliferation, inactivate α-SMA and reduce collagen production [67]. In concert, HGF counteracts ERK1/2 and JNK1 phosphorylation, decreases TGF-β and type I collagen gene expression [68,70] and promotes the apoptosis of activated HSCs and portal myofibroblasts [69], while inhibiting platelet-derived growth factor-B (PDGF-B) receptor docking.

In parallel to the decreased collagen production, fibrolysis is promoted by the HGF-mediated upregulation of matrix-metalloproteinases (MMPs) [71]. This could be confirmed in murine fibrosis models using carbon tetrachloride (CCl4) or dimethylnitrosurea (DMN), where the injection of platelet-rich plasma (PRP) significantly reduced hepatic fibrosis by increasing fibrolytic markers such as HGF and MMPs [70,71]. Although human platelets secrete less HGF than rodents [72], TPO receptor agonists stabilize liver functions and reduce liver fibrosis in patients with liver cirrhosis, HCV infection and CLD [73,74]. This treatment is particularly beneficial to patients with preceding thrombocytopenia [74,75]. Mechanistically, increased ECM degradation by MMP-9, reduced HSC activation and TGF-β as well as a decreased expression of the myofibroblast marker α-SMA were observed upon administration of TPO in an CCl4-induced mouse model of liver fibrosis [76] and in an rat partial hepatectomy model [77,78].

Contradicting the beneficial effects of platelets in liver fibrosis, platelet inhibition with aspirin is also protective [79,80]: in a cross-sectional analysis with CLD patients, aspirin was associated with lower incidences of liver fibrosis [80]. This correlation was validated by various other clinical [53,79,81] and pre-clinical studies, showing that the administration of aspirin reduces fibrotic and increased fibrolytic markers [82]. This indicates that platelets do not only bear anti-fibrotic functions but also accelerate fibrosis progression and activate HSC. Activated platelets aggravate liver fibrosis by releasing profibrotic molecules from their granules [83] and form microthrombi within the liver, especially near the fibrotic area [84]. Further, platelets seem to exacerbate local inflammation via leukocyte recruitment [85]. An important activator of HSC, which mediates the activation of downstream SMAD proteins, especially SMAD3, which promotes the transcription of type I and type III collagens, is TGF-β [86]. It is well known that platelets are major secretors of TGF-β [87], and hence the platelet-specific depletion of TGF-β1 decreases CCl4-induced liver fibrosis by reducing collagen synthesis and profibrotic signaling in HSCs [88].

Moreover, the release of PDGF-B by activated platelets promotes a continuous HSC activation, proliferation and migration [89], which subsequently causes myofibroblast transition [69,83,90,91]. Consequently, treatment with aspirin or a PDGF-B-specific antibody significantly improves liver fibrosis in mouse models [83,84]. Further, the platelet-derived chemokine CXCL4 mediates liver fibrosis by inducing proliferation, chemotaxis, and chemokine expression in HSCs, and CXCL4 serum levels correlate with the progression of liver fibrosis in HCV patients. In a CCl4 mouse model, the deletion of CXCL4 inhibited liver fibrosis and was associated with a decreased expression of profibrotic genes and a reduction of immune cell infiltration in the liver parenchyma [84].

Moreover, other platelet-stored factors, including vWF, 5-HT and sphingosine-1-phosphate (S1P), exert a pro-fibrotic effect: the depletion of vWF in mice with CCl4-induced fibrosis reduces the fibrotic area and improves liver function [92], and vWF could represent a useful biomarker for liver fibrosis and HCC in patients with HBV and HCV [93]. Additionally, 5-HT influences the activation and migration of HSC [94]. Antagonists of the 5-HT2A receptor inhibit HSC activation and facilitate their apoptosis, thereby propagating the resolution of hepatic fibrosis [95]. Moreover, the inhibition of the 5-HT2B receptor reduces fibrinogenesis and improves the liver function in a mouse fibrosis model [96].

Further, platelet-derived S1P signaling aggravates hepatic fibrosis via HSC activation and migration [90,97,98], and targeting the pathway or its receptor reduced fibrotic scars in cholestasis-induced liver injury [99].

In conclusion, the role of platelets in liver fibrosis is still incompletely understood. Platelets exhibit a dual role on liver fibrosis progression and resolution, as their granules contain pro- as well as anti-fibrolytic molecules. Discrepancies might occur due to the use of different models and methods as well as timing. Therefore, further studies are warranted to get a clear picture of platelet involvement in liver fibrosis.

## 7. Hepatocellular Carcinoma (HCC)

**Figure 6:**
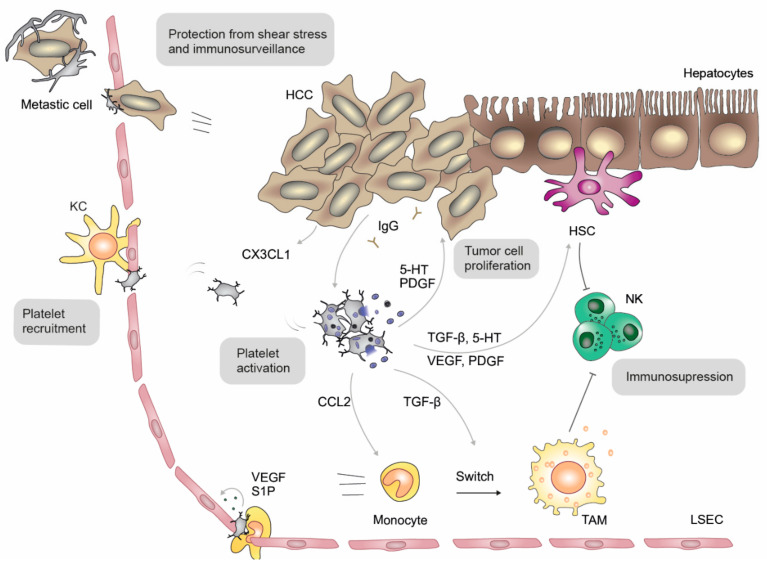
Platelets in hepatocellular carcinoma (HCC). During cancer progression, platelets are recruited by Kupffer cells (KC) and chemokine (C-X3-C motif) ligand (CX3CL1) release from cancer cells and subsequently become activated in the tumor environment (e.g., by immunoglobulins [Ig]). The release of their granule content promotes tumor cell proliferation and the activation of hepatic stellate cells (HSC). Moreover, the interaction with liver sinusoidal endothelial cells (LSEC) promotes tumor angiogenesis and facilitates the infiltration of leukocytes, which eventually develop into tumor-associated macrophages (TAM) that dampen immunosuppression by natural killer (NK) cells. Platelets promote tumor metastasis by protecting tumor cells from recognition by the immune system and from increased shear stress in various body fluids. Following mediators are involved: serotonin (5-HT), sphingosine-1-phosphate (S1P), platelet-derived growth factor (PDGF), transforming growth factor-*β* (TGF-*β*), vascular endothelial growth factor (VEGF) and CC-chemokine ligand 2 (CCL2).

Platelets play a central role in cancer development and are also involved in various steps of HCC development and progression (Figure 6) [100,101]. In a mouse model of NASH-induced HCC [9] and chronic immune-mediated hepatitis B [102], antiplatelet therapy with aspirin/clopidogrel or ticagrelor significantly reduced HCC development and progression, but clinical evidence is incomplete: platelet counts increase during the transformation from cirrhosis to HCC, and counts correlate with the HCC tumor stage patients. In a retrospective study with over 1000 patients suffering from unresectable HCC, thrombocytopenia was associated with increased survival and decreased metastatic potential [103]. Another study found a positive correlation between tumor size and platelet count [104], indicating a complex and highly regulated interplay between platelets and tumor cells, which has only been partially understood.

Platelets are recruited into the HCC tissue by tumor cell-derived CX3CL1 [105] and get activated. A unique tumor-specific feature is platelet activation by HCC-derived IgGs via FcγRIIa, which is upregulated in hyperreactive platelets of HCC patients [106]. Cytological smears from human HCC biopsies show that activated platelets cluster in close proximity to tumor cells [105,107,108] and adhere via GPIIb/IIIa, GPIb-IX-V and P-selectin receptors [108,109]. The inhibition of platelet activation with clopidogrel reduces the binding of platelets to hepatoma cells and prevents tumor growth [110]. Tumor-associated platelets act as a biological shield that protects tumor cells from recognition by immune cells [111]. Moreover, by the release of their granule content and by a direct contact with tumor cells, platelets facilitate tumor proliferation, trans-endothelial migration and epithelial-mesenchymal transition. Platelet-derived TGF-β downregulates the tumor suppressor gene KLF6, leading to enhanced tumor cell proliferation and cell cycle progression in vitro as well as enhanced tumor cell growth in vivo [112]. In vitro 5-HT binding to its receptor 5HTR2B promotes cell survival and proliferation via the inhibition of autophagy in a mTOR-independent process, and mice treated with 5HTR2B antagonists developed smaller tumors [113]. Similarly, mice lacking peripheral 5-HT showed a reduced incidence of HCC after CCl_4_ exposure. In human HCC biopsies, a correlation between HTR2B-positive tumor cells and tumor proliferation was observed [113]. 5-HT increases 5HTR2B expression, leads to a higher expression of the oncogene Yap via the 5HT-pERK-Yap axis [114] and plays a crucial role in tumor reoccurrence [115]. Platelet counts positively correlate with HCC recurrence or non-responders of chemotherapy, while counts from responders decreased upon HCC treatment [107]. Furthermore, high platelet counts correlate with an increased risk of developing extrahepatic metastasis [116]—likely due to platelet-mediated cell growth, tumor aggressiveness and migration [117]. Moreover, platelet-mediated metastasis is prevented by the inhibition of coagulation factor FVIII, which plays a role in platelet-cancer cell interaction [118]. However, beside their proliferating effects, platelets also promote HCC cell apoptosis [105].

Besides their direct interactions with cancer cells, platelets also affect the tumor microenvironment by recruiting leukocytes and by interacting with liver sinusoidal endothelial cells (LSECs) and HSCs, and therefore indirectly promote tumor growth and angiogenesis. Platelets foster LSEC proliferation and induce the production of interleukin-6 (IL-6) and the vascular endothelial growth factor (VEGF) [119], which upregulates hepatocyte proliferation and endothelial fenestrations [120]. Moreover, platelet-mediated HSC activation promotes an immunosuppressed environment in which tumor cells can thrive due to secreted cytokines and angiogenic growth factors [83,121,122].

Monocytes are recruited into the tissue by CCL2, VEGF and TGF-β. While VEGF and TGF-β are stored in platelets, the chemokine CCL2 was shown to bind to platelets and trigger monocyte arrest in vitro [123]. Apart from recruitment, platelet releasates were also shown to enforce a switch from monocytes to tissue resident M2 macrophages, which exhibit an anti-inflammatory phenotype and promote a pro-tumoral microenvironment by their immunosuppressive properties [124].

## 8. Liver Regeneration

**Figure 7:**
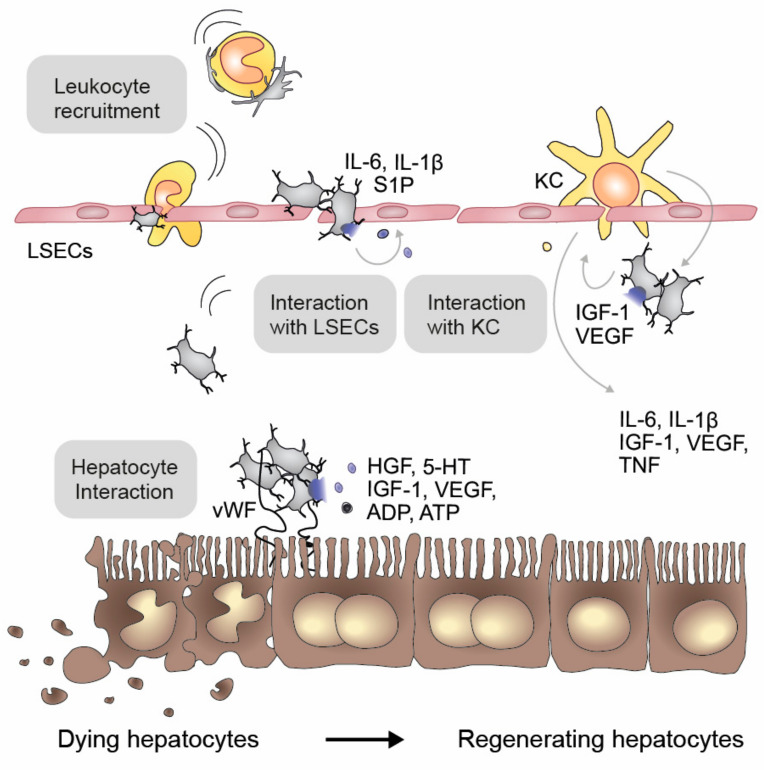
Platelets in liver regeneration. During liver regeneration, activated platelets interact with hepatocytes by binding to the von Willebrand factor (vWF) and by the release of their alpha and dense granule content, which contains hepatic growth factor, serotonin (5-HT), insulin growth factor-1 (IGF-1), vascular endothelial growth factor (VEGF) as well as adenosine diphosphate (ADP) and triphosphate (ATP). Due to the interaction of platelets with liver sinusoidal endothelial cells (LSECs) and the subsequent release of soluble mediators, leukocytes are recruited into the Space of Disse, where they act in concert with Kupffer cells (KC) to modulate liver regeneration. The following mediators are involved: interleukin-6 (IL-6), interleukin-1*β* (IL-1*β*), sphingosine-1-phosphate (S1P) and tumor necrosis factor (TNF).

Hepatocyte proliferation during liver regeneration is controlled by multiple extracellular signals. Some are directly mitogenic and others indirectly accelerate liver regeneration. Intracellular signaling pathways in hepatocytes occur within minutes after partial hepatectomy. However, the mechanisms triggering these pathways are incompletely understood—particularly in humans. Minutes after partial hepatectomy, platelets also accumulate in the space of Disse and get in direct contact with hepatocytes (Figure 7) [125,126], and recent evidence suggest that this process might be vWF-mediated [127]. This triggers platelet activation and the subsequent release of soluble mediators such as HGF, insulin-like growth factor (IGF-1) and PDGF, VEGF, 5-HT, ADP and ATP [128,129]. The initial vWF burst on the hepatocyte surface, followed by subsequent platelet accumulation [130], is essential for PI3K/AKT-dependent hepatocyte mitosis and an important initiator of liver regeneration [8,131,132,133,134]. Further fibrin(ogen) depositions act as a direct stimulus for liver regeneration, indicating that platelet and coagulation-stimulated regeneration pathways in the liver are interconnected [135].

Platelet counts and platelet transfusions are of clinical importance in liver regeneration, as low immediate postoperative platelet counts after liver resections are associated with delayed liver function and recovery, lower volumetric liver gain and more hepatic insufficiency, higher occurrence of liver dysfunction and long-term mortality [5,6,136]. Further, perioperative TPO levels potentially identify high-risk patients for liver dysfunction after hepatic resection [137], and TPO injections to increase platelet counts could be helpful in liver regeneration without fostering cancerous lesions, as demonstrated in a pilot study [74]. While the therapeutic efficacy of TPO seems limited in steatotic livers [138], inducing platelet counts might be a treatment option in other patients undergoing hepatectomy.

Mouse and rat partial hepatectomy models revealed that platelet transfusion accelerates liver regeneration already at early stages via PI3K/Akt and EK1/2 activation [125,139] and the reduction of antioxidant parameters [140], leading to an increase in hepatocyte mitosis and an elevated liver to body ratio [125,139,141]. However, exogenous platelet transfusions are not the only factor causing hepatocytes to reenter the cell cycle post hepatic resection; TPO injections also correlate with a faster liver regeneration, a higher mitotic index and increased Ki-67 activity in hepatocytes. These pro-regenerative effects of TPO are associated with elevated levels of HGF, IGF-1 and PDGF, and the activation of hepatic PI3K/AKT, ERK1/2 and STAT3 pathways [125,126,142,143]. Moreover, TPO administration has also been reported to stimulate liver regeneration indirectly, by inducing KC activation and LSEC proliferation, which in turn augments hepatocyte proliferation [126,142]. LSECs secrete mitotic substances, such as VEGF, HGF, IL-1 and IL-6, which potentially foster hepatocyte proliferation after a partial hepatectomy [144]. The direct contact of platelets with LSECs induces VEGF and S1P secretion from platelets, which could induce VEGF and IL-6 secretion by LSECs. It also suppresses their apoptosis and induces their LSEC proliferation [119]. LSEC-secreted IL-6 induces HGF production by HSCs and thereby hepatocyte proliferation [145]. Besides LSEC and HSC, platelets directly bind to KCs or indirectly stimulate them via the release of growth factors such as VEGF and IGF-1. KCs in turn induce platelet accumulation and foster their activation. KCs promote liver regeneration by secreting cytokines like IL-6, IL-1*β*, IGF-1 and tumor necrosis factor-α (TNF-α) [146], which activate proliferation-initiation pathways such as NF-κB and STAT3 [147]. The importance of KCs in liver regeneration becomes apparent in a KC depletion model, which delays liver regeneration in a TNF-α-dependent manner [126]. Platelets also contain anti-proliferative substances such as TSP-1. Hepatic microcirculation disturbances could potentially be responsible for selective α-granule release, which correlates with postoperative liver regeneration [8].

Although the importance of platelet-derived HGF is under debate due to its limited amount in human platelets, platelets indirectly increase HGF levels via the interaction with liver-resident cells such HSCs, LSECs and KCs [72]. For example, platelets secrete stromal derived factor-1 (SDF-1) and VEGF-A, which results in the angiocrine production of HGF and Wnt2 via Id1 activation [148]. Moreover, LSECs as well as hepatocytes are thought to internalize platelets [149], which results in the transfer of platelet RNA, which might then in turn stimulate hepatocyte proliferation in vitro [150].

Besides the direct or indirect delivery of growth factors and cytokines, platelet-derived 5-HT also induces liver regeneration. Preclinical studies revealed that 5-HT acts directly on hepatocyte proliferation and might be involved in the release of growth factors at the liver injury site, e.g., via IL-6 [151,152,153]. Furthermore, 5-HT is also an initiator of VEGF-dependent pathways in liver regeneration, thereby initiating neovascularization [154]. Patients with low platelet 5-HT before liver resection suffer from delayed hepatic regeneration, indicating that 5-HT levels represent a helpful clinical marker to predict postoperative liver dysfunction and clinical outcome [155]. While inappropriate plasma preparation might mask this association [156], the intake of selective serotonin reuptake inhibitors (SSRI) and serotonin noradrenalin reuptake inhibitors (SNRI) was associated with an adverse postoperative outcome after hepatic resection in a recent multicenter trial [45], suggesting that SSRI/SNRIs should be avoided perioperatively in these patients.

Platelets interact not only with liver cells but also with the immune system via the release of a multitude of chemokines and cytokines, including TGF-*β*, CCL5, CXCL8, CCL3, SDF-1 and CXCL4 [157]. Thus, the platelet-mediated accumulation of leukocytes could further explain the beneficial effect of platelets in liver regeneration [135,158,159].

However, further studies are warranted to unravel the full spectrum of platelets in liver regeneration, as all mentioned mechanisms might be affected by underlying liver diseases and might therefore differ within individual patients and underlying pathologies.

## 9. Conclusions and Outlook

During the last decade it became apparent that hemostasis and immunological processes are closely interconnected, and that platelets play a crucial role in liver diseases. However, due to their diverse functions and multiple cellular interactions, platelets represent a double-edged sword in liver pathologies.

Many liver diseases, ranging from NAFLD to HCC, are currently diagnosed by imaging techniques and tissue biopsy, which are more cost- and labour-intensive compared to less invasive blood-based liquid biopsies. Platelet-dependent parameters associated with these diseases might bear marker potential, and platelet-derived RNAs and proteins have the capability to serve as diagnostic and/or prognostic markers of liver diseases [160]. Especially for HCC, which is often diagnosed at advanced stages, there is an urgent need for biomarkers that allow early detection. The RNA repertoire of platelets changes during HCC progression [161,162], and their activation status is associated with HCC development and poor prognosis after treatment [163]. As platelets promote leukocyte infiltration into the steatotic liver of NAFLD patients, anti-platelet medication might help to prevent or ameliorate the progression of NAFLD into NASH and NASH-induced HCC. This is supported by clinical evidence that points toward a beneficial effect of aspirin [53]; however, further research is warranted to identify the crucial time window of treatment and to pinpoint the involved molecular mechanisms that guide activated platelets into the steatotic liver microenvironment. Moreover, in liver regeneration, platelet-related markers could predict the clinical outcome and could also potentially bear therapeutic potential, which is urgently needed, as currently no treatment exists for these patients.

Besides their theragnostic potential, recent data indicate that platelets may also serve as a therapeutic vehicle, enabling optimized and controlled anti-tumor therapy release or tissue regeneration processes. Platelet-based drug delivery was shown to be superior to nanoparticle systems and to improve drug tissue enrichment while reducing adverse effects in murine models [164,165].
